# Liquid–liquid phase separation driven compartmentalization of reactive nucleoplasm

**DOI:** 10.1088/1478-3975/abc5ad

**Published:** 2021-01-07

**Authors:** Rabia Laghmach, Davit A Potoyan

**Affiliations:** 1 Department of Chemistry, Iowa State University, Ames, IA 50011, United States of America; 2 Department of Biochemistry, Biophysics and Molecular Biology, Iowa State University, Ames, IA 50011, United States of America; 3 Bioinformatics and Computational Biology program, Iowa State University, Ames, IA 50011, United States of America

**Keywords:** phase separation, gene regulation, nucleoplasm, liquid–liquid phase separation, eukaryotic nucleus, nonequilibrium

## Abstract

The nucleus of eukaryotic cells harbors active and out of equilibrium environments conducive to diverse gene regulatory processes. On a molecular scale, gene regulatory processes take place within hierarchically compartmentalized sub-nuclear bodies. While the impact of nuclear structure on gene regulation is widely appreciated, it has remained much less clear whether and how gene regulation is impacting nuclear order itself. Recently, the liquid–liquid phase separation emerged as a fundamental mechanism driving the formation of biomolecular condensates, including membrane-less organelles, chromatin territories, and transcriptional domains. The transience and environmental sensitivity of biomolecular condensation are strongly suggestive of kinetic gene-regulatory control of phase separation. To better understand kinetic aspects controlling biomolecular phase-separation, we have constructed a minimalist model of the reactive nucleoplasm. The model is based on the Cahn–Hilliard formulation of ternary protein–RNA–nucleoplasm components coupled to non-equilibrium and spatially dependent gene expression. We find a broad range of kinetic regimes through an extensive set of simulations where the interplay of phase separation and reactive timescales can generate heterogeneous multi-modal gene expression patterns. Furthermore, the significance of this finding is that heterogeneity of gene expression is linked directly with the heterogeneity of length-scales in phase-separated condensates.

## 1. Introduction

Phase separation is a fundamental mechanism for the emergent order in an ordinary and biological matter [1, 2]. Recently, phase separation of biomolecules has also become a cornerstone physical mechanism for understanding the intracellular organization [2–4]. A wide range of membrane-less compartments are found to form through biomolecular phase separation, including nucleoli [5–7], stress granules [8–10], chromatin domains [11–13] and transcriptional centers [14, 15]. The primary components driving the intra-cellular phase separation are proteins and nucleic acids with multi-valent interaction centers [16, 17].

The stickers-and-spacers framework has emerged as a viable model explaining the existence of a broad class of sequence encoded driving forces of disordered proteins which serve as nucleating centers for biomolecular condensates [3, 18]. These newly appreciated abilities of proteins and nucleic acids for forming large-scale liquid bodies is offering fresh avenues for understanding mechanisms of the coordinated action of biomolecules in gene regulation and cellular organization that go beyond single-molecule action. It is well known that eukaryotic nuclei are rich in disordered proteins linked with transcription and chromatin architecture reorganization activities [19, 20]. There have been several proposals of the functional roles that may include catalysis of biochemical reactions, noise buffering and inducing ultra-sensitive signals [21–23]. However, understanding the mechanistic picture that links phase separation to the functional gene regulatory processes inside the nucleus has remained elusive [22] due to heterogeneous, multiscale, and non-equilibrium nature of the nuclear environment [24].

**Figure 1.**
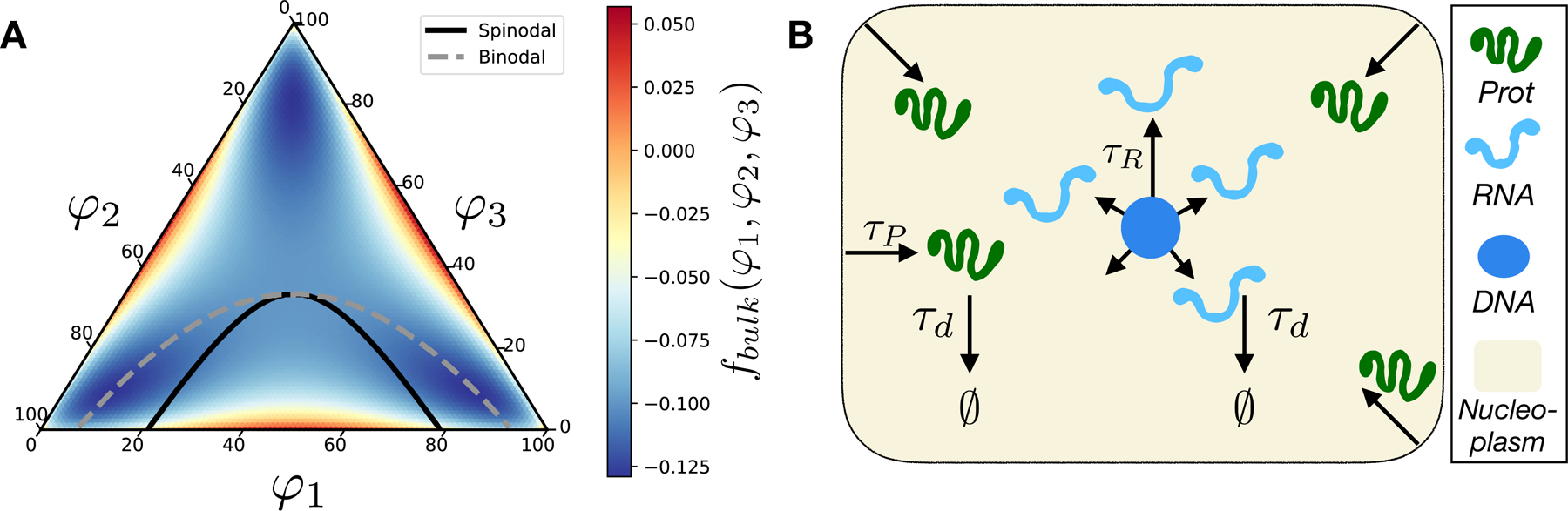
(A) Phase diagram of the symmetric ternary protein–RNA–nucleoplasm mixture and the bulk free energy governing the solution thermodynamics *f*_bulk_(*φ*_1_, *φ*_2_, *φ*_3_) for *χ* = 3. The solid-line and dash-line correspond to the spinodal and binodal curves, respectively. (B) The schematic of the minimal reactive nucleoplasm model. Shown are the main reactive components (protein, RNA, DNA-seed, nucleoplasm reservoir), the corresponding reactive processes involving each components as well as their spatial generation profiles.

In this work, we propose a minimal model of a reactive nucleoplasm (figure 1) with an objective to illustrate, in a proof of principle manner; (i) how spatially resolved non-equilibrium reactive enevents generate qualitatively distinct from equilibrium phase behavior and (ii) how the interplay of various kinetic timescales in the system impacts gene expression patterns. The minimal reactive nucleoplasm model consists of a ternary solution filled with incompressible fluid consisting of ‘active’ protein, RNA components, and ‘passive’ nucleoplasmic buffer. The mathematical formulation of the model is based on a generic ternary diffuse interface model of one-step transcription/translation and phase-separation inside the nucleoplasm. The model resolves the formation and dissolution of protein–RNA droplets as well as reactive events of influx/creation and out-flux/degradation of proteins and RNA. By an extensive set of simulations exploring the interplay of timescales in the system, we found a broad kinetic regime dominated by length-scale heterogeneity of phase-separated droplets which correspond to heterogeneous gene expression patterns.

Various simple mathematical models have been used to explore generic aspects of equilibrium and non-equilibrium phase-separation as well as the physical properties of resulting condensates [25–33]. The most relevant to the present contribution are the work by Berry *et al* [29] , Tang and Yang [33], and Glotzer [32], where authors have considered a binary and ternary fluid model of nucleoplasm, which couples phase separation with first-order exchange among soluble and insoluble components. Authors have simulated different stages of spinodal decomposition and explored its impact on Ostwald ripening of droplets. It was shown that kinetics of the RNA flux accelerates the ripening of droplets, thereby showing a link between the thermodynamics of phase separation and kinetics of droplet formation. Other recent notable studies worth highlighting here include the work by Yamamoto *et al* [30], which have studied the non-spatial model of architectural RNA phase separation coupled with non-equilibrium production. Finally, an important theoretical framework by Ilker and Joanny [34] establishes and equivalence of phase-separation kinetics with the Cahn–Hilliard effective temperature.

Some of the key distinctions of the present model from previous studies include (i) spatially resolved formulation of gene expression and phase separation (ii) explicit connection of phase-separated patterns with transcription and translation models of gene expression (iii) exploring the impact of dynamic turnover in RNA–protein–nucleoplasm ternary diagram on global patterning. Thus, the present work clarifies as a first step the dual nature of nuclear order and gene expression, and provides a useful theoretical framework for understanding equilibrium and non-equilibrium origins of intra-nuclear patterning.

## 2. Minimal reactive nucleoplasm model

In order to explore the kinetics of phase separation of RNA–proteins–nucleoplasm reacting mixture, we use a local thermodynamic approach that describes the phase separation of a ternary mixture based on the Flory–Huggins model [35, 36] for polymer solutions coupled with chemical reaction-diffusion equations (figure 1). In this section, we describe the mathematical formulation of the model of a ‘minimal reactive nucleoplasm’: where the liquid–liquid phase separation of single RNA and protein components is coupled with reactive events of transcription, translation, and degradation.

A minimal one-step model of independent, unregulated transcription and translation [37] is used to set the lifetimes of protein and RNA components comprising the nucleoplasmic milieu defined by the following the chemical reactions:

(1) $$\begin{array}{l} \emptyset \overset{k_{\text{R}}}{\to }\text{R}\overset{k_{\text{d}}}{\to }\emptyset ,\\ \emptyset \overset{k_{\text{P}}}{\to }\text{P}\overset{k_{\text{d}}}{\to }\emptyset , \end{array}$$

where the *k*_R_, *k*_P_ and *k*_d_ are the rate coefficients for transcription, translation and degradation, respectively; The ∅ symbol is used to denote the degradation of RNA and protein. The kinetics of phase separation of ternary reactive protein–RNA–nucleoplasm mixture is given by the following set of reaction–diffusion equations [32]:

(2) $$\frac{\partial \varphi_{1}}{\partial t}=\nabla \left(M_{1}(\{\varphi_{i}\})\nabla \left(\frac{\partial F[\{\varphi_{i}\}]}{\partial \varphi_{1}}\right)\right)\;\;\;+f_{1}\left(r\right)\left[k_{\text{R}}(1-\varphi_{1}-\varphi_{2})-k_{\text{d}}\varphi_{1}\right]$$

(3) $$\begin{array}{l} \frac{\partial \varphi_{2}}{\partial t}=\nabla \left(M_{2}(\{\varphi_{i}\})\nabla \left(\frac{\partial F[\{\varphi_{i}\}]}{\partial \varphi_{2}}\right)\right)\\ \;\;+f_{2}\left(r\right)\left[k_{\text{P}}(1-\varphi_{1}-\varphi_{2})-k_{\text{d}}\varphi_{2}\right], \end{array}$$

where *φ*_*i*_(*i* = 1, 2, 3) are the order parameter variables associated with the local concentration of *i*th-component forming the mixture, *M*_*i*_ are their corresponding mobility coefficients, and *F* is the free energy functional describing the thermodynamics of ternary mixture. The total local density in the system is considered constant, respecting the incompressibility condition expressed as: $\sum_{i=1}^{3}\varphi_{i}=1$. The indices 1, 2, and 3 denote RNA, protein, and nucleoplasm, respectively. The function *f*_1_(*r*) and *f*_2_(*r*) are used to define the spatially heterogeneous distribution of components mimicking RNA expression inside nucleus and protein translation and flow into nucleus. The RNA is created in the center of nucleoplasm thereby mimicking a transcriptional process [38], herein we used the function defined as *f*_1_(*r*) = exp(−(*r* − *r*_c_)^2^*/a*) where *r* is the distance from the center of the domain located at *r*_c_. The coefficient a is a localization lengthscale which is set to *a* = 1. The protein component is flown into the nucleus from the nuclear boundaries thereby mimicking translation [38], herein we used the function defined as *f*_2_(*r*) = 1 on the domain boundary *∂*Ω and 0 otherwise. Heterogeneous mobility is an interesting aspect of nucleoplasm [39], however, and is undoubtedly deserving of a separate investigation. For simplicity, we assume the mobility coefficients for all components to be constant *M*_1_({*φ*_*i*_}) = *M*_2_({*φ*_*i*_}) = *M*. The free energy functional taken in this model is based on the Flory–Huggins energy formulation of the ternary mixture system [40, 41], which is given by:

(4) $$F[\left\{\varphi_{i}\right\}]=\int \text{d}\Omega \left[f_{\text{bulk}}\left(\varphi_{1},\varphi_{2},\varphi_{3}\right)+\sum_{i}\frac{\kappa_{\varphi i}}{2}|\nabla \varphi_{i}{|}^{2}\right]$$

with

$$f_{\text{bulk}}\left(\varphi_{1},\varphi_{2},\varphi_{3}\right)=\sum_{i}\frac{\varphi_{i}}{N_{i}}\ln\left(\varphi_{i}\right)+\sum_{i\neq j}\chi_{ij}\varphi_{i}\;\varphi_{j},$$

where *f*_bulk_(*φ*_1_, *φ*_2_, *φ*_3_) is the local free energy density, and the square-gradient coefficients *κ*_*φi*_ are positive constants controlling the interfacial free energies. *χ*_*ij*_ are the interaction parameters between species *i*–*j*, and *N*_*i*_ is the degree of polymerization.

We note here that there also exist other mathematical formulations of the local free energy for describing the phase separation of ternary polymeric mixtures. For instance, the generalized multiwell potential used by Yang *et al* [42] to study multiphase systems where the local free energy density is the summation of all the double-well potential for each phase-field variable complemented by a polynomial term that connects all the variables altogether.

The dynamic equations are brought to a dimensionless form, which reveals the essential time- and length-scales of the problem. Let us introduce *l*, the characteristic length of the system, and *τ* is the characteristic time. The dimensionless form of the kinetic equations yields four dimensionless parameters that control the dynamics of phase-separation where components are undergoing chemical reactions. The *τ*_D_*/τ* = *l*^2^*/*(*M* × *τ* ) = 1 is the ratio between diffusion timescale and characteristic time which is fixed to be a unit. The *τ/τ*_T_ = *τ* × *k*_T_ is the ratio between characteristic time and time-scale associated with transcription. The *τ/τ*_P_ = *τ* × *k*_P_ is the ratio between characteristic time and time-scale associated with protein formation. The *τ/τ*_d_ = *τ* × *k*_d_ is ratio between characteristic time and time-scale associated with degradation. To solve the dynamic equations (2) and (3) in the dimensionless form, we use a fully implicit finite element C++ library from the multiphysics object-oriented simulation environment (MOOSE) [43]. The simulations were performed on a rectangular domain of dimensions *L*_*x*_ × *L*_*y*_ : (50 × 50), with periodic boundary conditions. A quadrilateral element QUAD4 with four nodes was used for domain meshing with the refinement. The total number of elements used for the fine mesh is 10 000. The time step of integration Δ*t* is fixed at 0.05 (a.u.). In this work, we only consider the case of a symmetric ternary mixture undergoing a chemical reaction with *χ*_12_ = *χ*_23_ = *χ*_13_ = 3 that ensures phase-separation of protein–RNA droplets in the absence of chemical reactions *χ > χ*_c_, where *χ*_c_ denote the critical point. We also assumed that the chemical reaction takes place at the comparable scales with the phase separation process. Near the critical point *χ*c, the mean-field Flory–Huggins free energy can be approximated through Taylor expansion via Landau form [32]. It is important to construct representative free energy functional that accounts for fluctuations of the phase-field variables that are dominant near the critical point. To express the critical behavior a new formulation of the free energy of mixing renormalized by the spatial variation has been proposed by Yamamoto *et al* [44, 45]. The chain lengths *N*_*i*_, or degree of polymerization, are assumed to be the same: *N*_1_ = *N*_2_ = *N*_3_ = 1. It is noted here that the entropic term of the local free energy is proportional to the inverse polymeric lengths *N*_*i*_. As a consequence of the reduction of entropy for long-chain lengths, the phase diagrams and the spinodal curve will be modified in the way to increase the separating-phase region of the phase diagram. In this case, a small variation of the interaction parameter between species will lead easily to phase separation. The impact of phase-separation of small and large chains such as genomic regions and proteins/RNA could be an interesting study to address in future investigations. The other parameters of simulations were set to *κ*_*∅*1_ = *κ*_*∅*2_ = 0.031 25. The initial configuration of phase-field variables is generated by *φ*_*i*_(*r*) = ⟨*φ*_*i*_⟩ + *δφ*_*i*_, where ⟨*φ*_*i*_⟩ is an initial average concentration associated with *φ*_*i*_, and *δφ*_*i*_ is a small random perturbation amplitude. For all simulations presented here, the initial average of species *i* are set to ⟨*φ*_1_⟩ = 0.3 and ⟨*φ*_2_⟩ = 0.09, with *δφ*_1_ ∈ [−0.05, 0.05] and *δφ*_2_ ∈ [−0.01, 0.01].

## 3. Results

**Figure 2.**
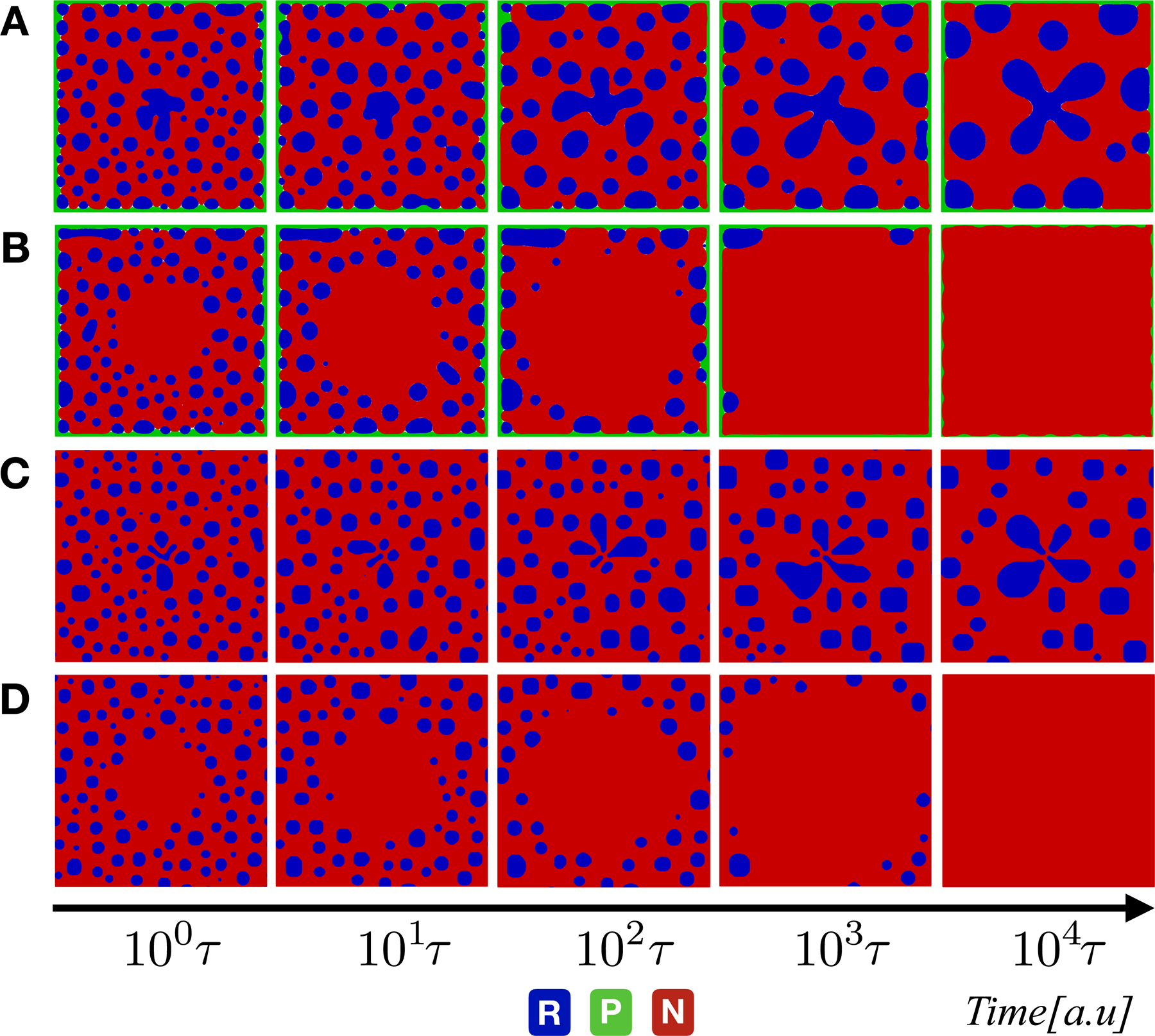
Evolution of phase-field variables *φ*_*i*_ for three phase mixture undergoing the chemical reaction with a degradation time-scale fixed at the same of diffusion (*τ*_d_ = *τ* ). From up to bottom: snapshots corresponding to simulation results with (A) *τ*_R_ = *τ*_P_ = *τ*; (B) *τ*_R_ = 100*τ*_P_ = 100*τ*; (C) *τ*_P_ = 100*τ*_R_ = 100*τ* ; and (D) *τ*_R_ = *τ*_P_ = 100*τ* . The color code in blue, green, and red indicates the RNA, protein, and nucleoplasm regions, respectively.

**Figure 3.**
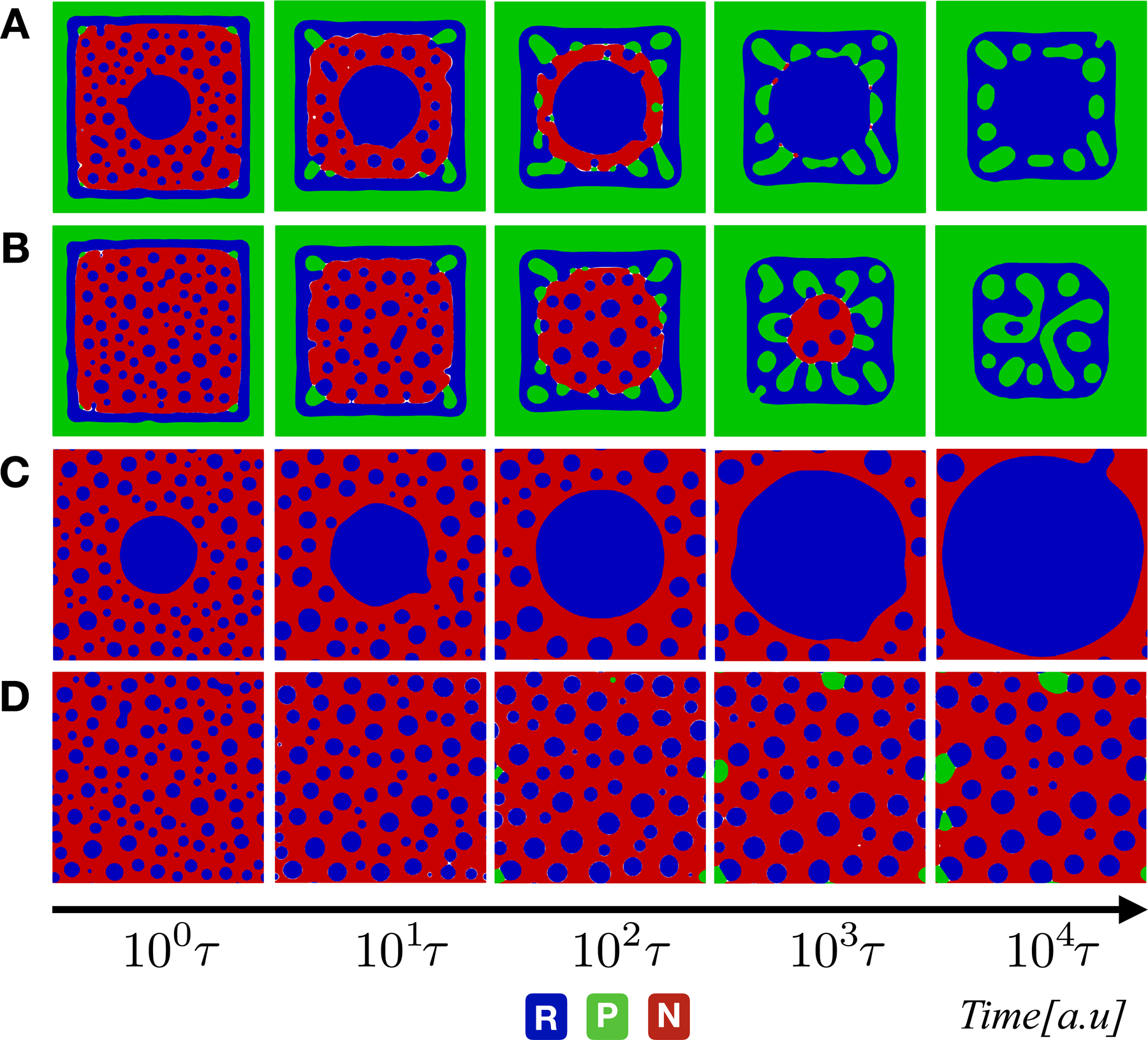
Evolution of phase-field variables *φ*_*i*_ for three phase mixture undergoing the chemical reaction with a degradation time-scale fixed at the same of diffusion (*τ*_d_ = 10*τ*). From up to bottom: snapshots corresponding to simulation results with (A) *τ*_R_ = *τ*_P_ = *τ* ; (B) *τ*_R_ = 50*τ*_P_ = 50*τ*; (C) *τ*_P_ = 100*τ*_R_ = 100*τ* ; and (D) *τ*_R_ = *τ*_P_ = 50*τ* . The color code in blue, green, and red indicates the RNA, protein, and nucleoplasm regions, respectively.

**Figure 4.**
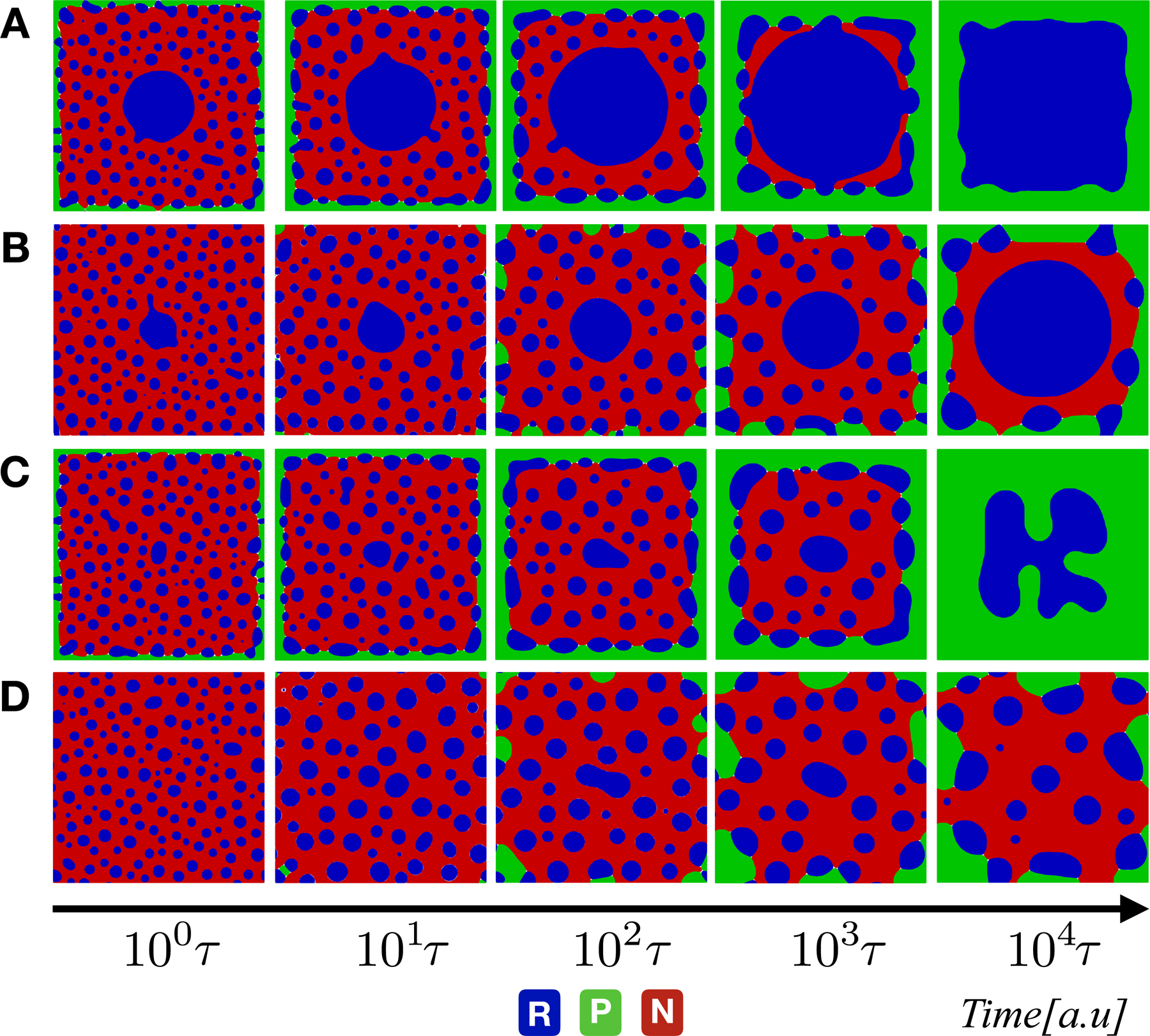
Evolution of phase-field variables *φ*_*i*_ for three phase mixture undergoing the chemical reaction with a degradation time-scale fixed at the same of diffusion (*τ*_d_ = 100*τ* ). From up to bottom: snapshots corresponding to simulation results with (A) *τ*_P_ = 10*τ*_R_ = 10*τ*; (B) *τ*_R_ = 10*τ*_P_ = 50*τ*; (C) *τ*_R_ = 50*τ*_P_ = 10*τ*; and (D) *τ*_R_ = *τ*_P_ = 100*τ*. The color code in blue, green, and red indicates the RNA, protein, and nucleoplasm regions, respectively.

Here we report the findings obtained by analyzing the results of simulations with a minimal reactive nucleoplasm model. We have organized the results in three broad kinetic regimes, which are described by three orders of magnitude in degradation time-scale *τ*_d_ = *τ*, 10*τ* , 100*τ* with respect to the diffusion time-scale *τ* . These time-scales correspond to different rates of turnover of nucleoplasmic components from fast to slow. For each regime, we have carried out extensive grid-based kinetic parameter sweeps exploring the coupling of transcriptional and transnational time-scales on the backdrop of fixed phase-separating free energy landscape of RNA–protein–nucleoplasm components. Despite the incredible simplicity of the minimal reactive nucleoplasm model, the time course of simulations (figures 2–4) has revealed a non-trivial patterning of nucleoplasm which are a dramatic departure from equilibrium thermodynamics of ternary phase separation in the absence of spatially non-uniform reaction–diffusion.

We start by investigating the fast dynamical regime corresponding to *τ*_d_ = *τ* . Figure 2 shows the spatial and temporal evolution of RNA–protein components in a reactive nucleoplasm environment. Four interesting cases are highlighted in figure 2, corresponding to different time-scale separation between translation *τ*_P_ and transcription *τ*_R_ processes. When *τ*_P_ = *τ*_R_ = *τ* we observe rapid coarsening dynamics (droplet growth kinetics) and emergence of RNA droplets with stable ‘protein front’ by which we refer to the formation of protein layer surrounding nucleoplasm domain edges [figure 2(A)]. Coarsening dynamics for either protein front or RNA droplets are also observed for fast translation/slow transcription or slow translation/fast transcription, respectively [figures 2(B) and (C)]. Naturally, it is expected that the more rapid degradation timescales for RNA and protein lead to the disappearance of both RNA droplets and protein fonts [figure 2(D)]. Thus, we can conclude that in the fast dynamical regime, nucleoplasm patterns are entirely set by the kinetic parameters with thermodynamic free energy landscape taking the back seat.

The formation of RNA–protein patterns is more intricate in the intermediate dynamical regime corresponding to *τ*_d_ = 10*τ* (figure 3). In the figure 3, we show the patterns arising from competing transcription, translation, and degradation with different transcription and translation timescales. When both transcription and translation have comparable time scales [figure 3(A)] to that of diffusion, we find a rapid progression of protein front on the one hand and a rapid RNA seed droplet growth at on the other. In this regime, once the nucleoplasm reaches a non-equilibrium steady state, droplet patterning is now becomes dictated by the thermodynamics of Flory–Huggins interaction parameters, which set the extend of mixing between protein and RNA components. When both the transcription and translation have the same time scale but are now significantly slower than diffusion [figure 3(D)], then the system displays heterogeneity in protein RNA droplet distribution. The situation with faster transcription and the faster translation is predictable: [figures 3(B) and (C)] the system tends to increase in protein front and RNA nucleating center, respectively. We have only highlighted the most interesting cases for each dynamical regime. The simulation results for the full set of time-scales are presented in the supporting information (https://stacks.iop.org/PB/18/015001/mmedia) figure S1–S10. The tempo ral evolution of average concentration for different kinetic regimes with *τ*_R_ = *τ*_P_ = 100*τ* is shown in figure S14.

We now turn to the analysis of the slow dynamical regime corresponding to a degradation timescale *τ*_d_ = 100*τ* . In this regime, we once again high-light four interesting cases [figures 4(A)–(D)]. When the system has comparable timescales for transcription, translation, and diffusion *τ*_P_ = *τ*_R_ = *τ*, the nucleoplasmic component gets eliminated, and the system rapidly reaches a balance between protein front and RNA seed. Slowing down the translation *τ*_P_ = 100*τ*_R_ = 100*τ* by two orders of magnitude leads to a non-trivial patterning with the RNA seed, nucleoplasm and RNA droplets all con-existing at a non-equilibrium steady-state. On the other hand, slowing down the transcription [figure 4(C)] leads to a steady-state with significantly downsized RNA-seed at the expense of the peripheral protein front. Slowing down both transcription and translation [figure 4(D)] leads to a non-trivial patterning with protein front, RNA droplets, and nucleoplasmic environment, but this time with no dominant RNA-seed.

**Figure 5.**
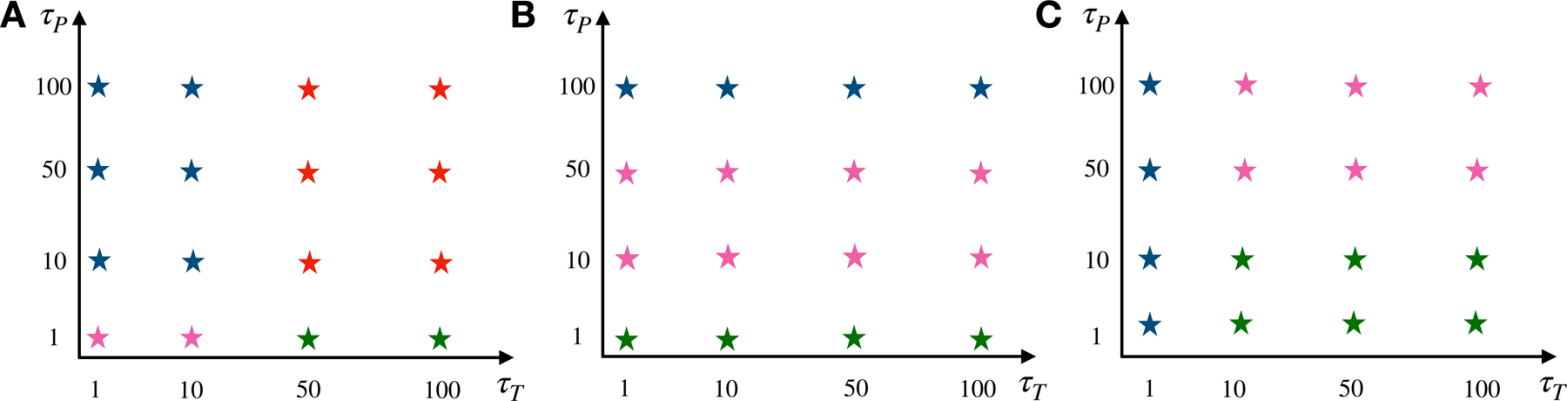
Phase diagram: phase diagram showing the dominant steady-state phase and summarizing various patterns that arise at three kinetic regimes (A) *τ*_d_ = *τ*; (B) *τ*_d_ = 10*τ*; (C) *τ*_d_ = 100*τ*). The blue star indicates that the RNA domain becomes the dominant phase in the long timescale limit. The green star means that the proteins-droplets become the dominant phase in the long timescale limit. The pink star indicates three coexisting phases of the ternary mixture. In contrast, the red star indicates that RNA–proteins turn totally into the nucleoplasm.

To quantify the emergence of different droplet patterns, we have summarized the phase behavior of a reactive nucleoplasm model via. Global kinetic phase diagram, figure 5. Here one can see a global picture of how the interplay of kinetic timescales is favoring uniform vs binary vs ternary phases.

**Figure 6.**
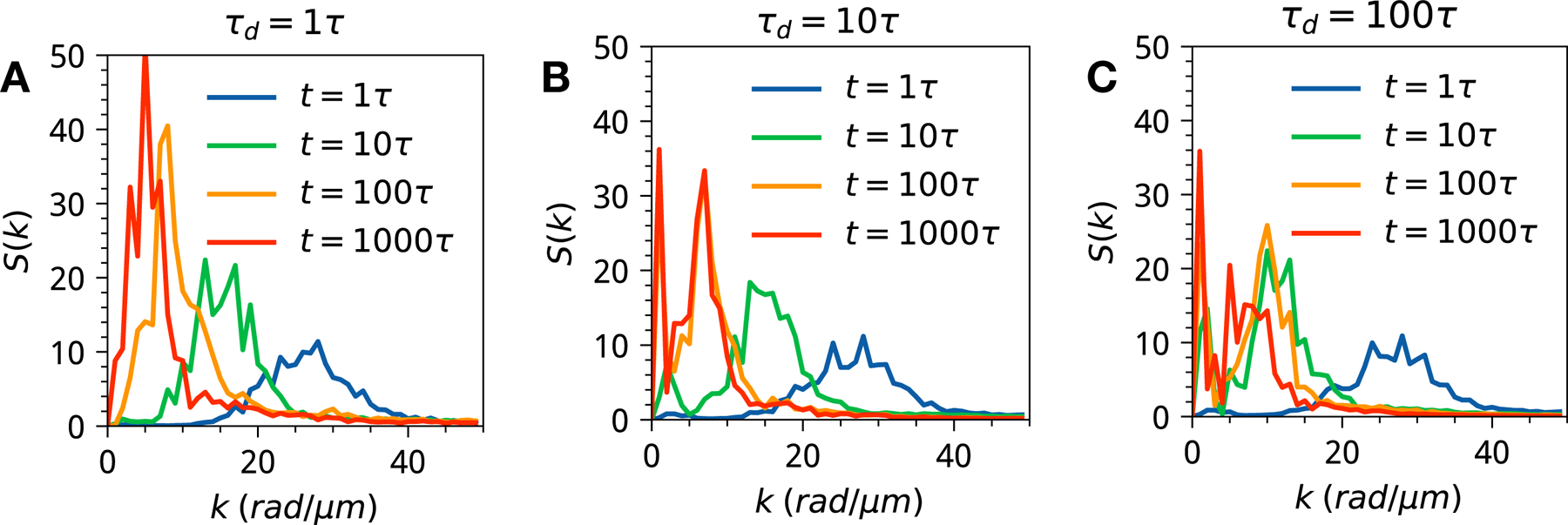
The dynamic structure factors for the three representative kinetic regimes with significant time-scale disparity between transcription and translation *τ*_P_ = 100*τ*_R_ (see supporting information for all the results). The inset shows the scaling and power law exponent. The three panels stand for three dynamical regimes: (A) *τ*_d_ = *τ* (B) *τ*_d_ = 10*τ* (C) *τ*_d_ = 100*τ*.

In order to quantify the length-scales of emergent patterns, we have analyzed the azimuthally averaged dynamic structure factors *S*(*k*, *t*) = *∫* d*k*_Ω_*S*(**k**, *t*) associated with each dynamical regime (see figure 6 and supporting information, figure S11–S13). Analyzing dynamic structure factors reveals characteristic length-scales of protein/RNA droplets as well as emergent dynamical heterogeneity manifesting in the fast or slow coarsening of the droplets. The computed structure factors show a broad range of kinetically controlled states where one has bi-modality of RNA (or protein) components. This bimodality or more broadly heterogeneity of distribution is directly linked to the heterogeneity of droplet sizes and shapes that one can quantify from simulation images (figures 3 and 4). Multi-modal gene expression is a feature often linked with phenotypic heterogeneity and which has been mostly explained by citing the underlying non-linear dynamics of dichotomous switching noise in a spatially uniform master equation formalism [46–48]. The results of figure 6 clearly show that transcriptional heterogeneity can originate purely from the spatially non-uniform nature of gene expression, which is being modulated by timescales of phase separation, transcription, and translation.

**Figure 7.**
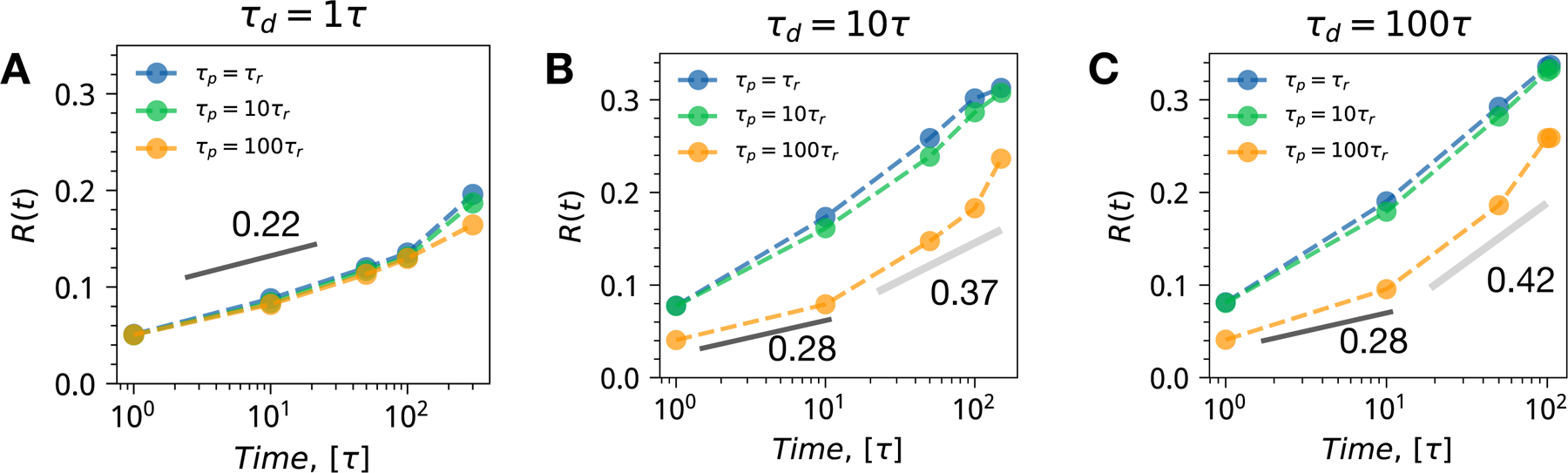
The dominant length-scale patterns for the three representative kinetic regimes. Each panel shows a combination of transcription and translation time-scales sorted with an increasing time-scale disparity from blue to green to orange. (A) *τ*_d_ = *τ* (B) *τ*_d_ = 10*τ* (C) *τ*_d_ = 100*τ*.

Finally, we have computed the length scale of patterns summarizing transitional heterogeneity in three kinetic regimes of the minimal reactive nucleoplasm model (figure 7). The length-scales patterns are quantified by using pre-computed azimuthally averaged dynamic structure factors $R\left(t\right)=\frac{\int \text{d}kS\left(k,t\right)k^{-2}}{\int \text{d}kS\left(k,t\right)k^{-1}}$ [49]. The length-scale patterns *R*(*t*) quantify the dominant length-scales which emerge during the time-evolution of a reactive nucleoplasm. Analyzing the evolution of length-scale patterns for three dynamical regimes, we see clearly that for the fast dynamical regime, there is only one dominating length-scale, which is dictated by the rapid degradation kinetic timescale. For the intermediate and slow dynamical regimes, however, we find heterogeneity of length-scales, which emerges from the disparity in transcription and translational timescales. We note that this heterogeneity has both structural and dynamical manifestations, as one can see by analyzing the power low of length-scale patterns *R*(*t*) ∼ *At*^*α*^. We find two exponents, one which is characteristic for the early coarsening stage (*α* ∼ 1*/*3) and second (*α* ∼ 3*/*8) for the later accelerated phase separating evolution toward a steady state. We note that power laws in the dynamical variables of the nucleoplasmic environment have been detected and characterized in a large number of experiments [39, 50]. These power-law dependencies, however, are hard to disentangle in terms of distinct contributions since their origin may come from any combination of phase-separation, polymeric effects, confinement, and non-equilibrium motorized activities in the nucleus. In this work, we have only managed to scratch at the surface of the fascinating dynamical patterning potential of the active nucleoplasmic environment. For future studies, it would be interesting to investigate the impact of differential mobility, hydro-dynamical coupling, and as well as investigate gene expression beyond a simple one-step model of unregulated reactions that have not been done in the present contribution.

## 4. Conclusion

Recent *in vitro* experiments with binary and ternary protein and RNA mixtures [51–53] have shown the rich complexity of phases which can emerge through liquid–liquid phase separation via modulation of stoichiometry of components and point mutations. These experiments show potentially novel regulatory strategies which when combined with non-equilibrium cellular processes such as transcription and translation could produce coordinated gene regulatory and signaling actions.

To this end, in the present contribution we introduce a minimal reactive nucleoplasm model combining spatially dependent transcription and translation to liquid–liquid phase separation of RNA and protein components embedded in the back-drop of passive nucleoplasm buffer. We use the minimal reactive-nucleoplasm model to cleanly dissect how the interplay of transcription, translation, and degradation time scales couples with the liquid–liquid phase separation of RNA and protein components in a model ternary solution. By carrying out extensive grid-based sweeps of kinetic parameter space, we uncover various non-trivial patterning and length-scale heterogeneity compared to the classic Flory–Huggins thermodynamic picture for ternary polymeric solutions. Our central finding is the existence of a broad kinetic regime characterized by a slow turnover of components and timescale disparity between transcription and translation under which a phase separating system can display bi-modal distribution. The significance of this finding is that the observed heterogeneity of gene expression is linked directly with the heterogeneity of length-scales in phase-separated condensates. The main findings and the minimal reactive nucleoplasm model thus establishes a useful framework with which one can further elucidate the emergence of nucleoplasm patterns and phenotype heterogeneity from first principles modeling of phase-separation and reaction–diffusion processes.

## Supplementary Material

Supplementary Information

## References

[1] Muñoz MA (2018). Colloquium: criticality and dynamical scaling in living systems. Rev. Mod. Phys.

[2] Berry J, Brangwynne CP and Haataja M 2018 Physical principles of intracellular organization via active and passive phase transitions Rep. Prog. Phys 81 046601 29313527 10.1088/1361-6633/aaa61e

[3] Choi J-M, Holehouse AS and Pappu RV 2020 Physical principles underlying the complex biology of intracellular phase transitions Annu. Rev. Biophys 49 107–33 32004090 10.1146/annurev-biophys-121219-081629 PMC10715172

[4] Dignon GL, Best RB and Mittal J 2020 Biomolecular phase separation: from molecular driving forces to macroscopic properties Annu. Rev. Phys. Chem 71 53–75 32312191 10.1146/annurev-physchem-071819-113553 PMC7469089

[5] Latonen L (2019). Phase-to-Phase with nucleoli—stress responses, protein aggregation and novel roles of RNA. Front. Cell. Neurosci.

[6] Frottin F 2019 The nucleolus functions as a phase-separated protein quality control compartment Science 365 342–7 31296649 10.1126/science.aaw9157

[7] Feric M, Vaidya N, Harmon TS, Mitrea DM, Zhu L, Richardson TM, Kriwacki RW, Pappu R V and Brangwynne C P 2016 Coexisting liquid phases underlie nucleolar subcompartments Cell 165 1686–97 27212236 10.1016/j.cell.2016.04.047 PMC5127388

[8] Van Treeck B, Protter DSW, Matheny T, Khong A, Link CD and Parker R 2018 RNA self-assembly contributes to stress granule formation and defining the stress granule transcriptome Proc. Natl Acad. Sci. USA 115 2734–9 29483269 10.1073/pnas.1800038115 PMC5856561

[9] Molliex A, Temirov J, Lee J, Coughlin M, Kanagaraj AP, Kim HJ, Mittag T and Taylor JP 2015 Phase separation by low complexity domains promotes stress granule assembly and drives pathological fibrillization Cell 163 123–33 26406374 10.1016/j.cell.2015.09.015 PMC5149108

[10] Riback JA, Katanski CD, Kear-Scott JL, Pilipenko EV, Rojek AE, Sosnick TR and Drummond DA 2017 Stress-triggered phase separation is an adaptive, evolutionarily tuned response Cell 168 1028–40 28283059 10.1016/j.cell.2017.02.027 PMC5401687

[11] Zhang Y and Kutateladze TG 2019 Liquid–liquid phase separation is an intrinsic physicochemical property of chromatin Nat. Struct. Mol. Biol 25 1085–6 10.1038/s41594-019-0333-8 PMC11514442 31695191

[12] Palikyras S and Papantonis A 2019 Modes of phase separation affecting chromatin regulation Open Biol 9 190167 31615334 10.1098/rsob.190167 PMC6833219

[13] Laghmach R, Di Pierro M and Potoyan DA 2019 Mesoscale liquid model of chromatin recapitulates nuclear order of eukaryotes Biophys. J 118 2130–40 31623887 10.1016/j.bpj.2019.09.013 PMC7202933

[14] Hnisz D, Shrinivas K, Young RA, Chakraborty AK and Sharp PA 2017 A phase separation model for transcriptional control Cell 169 13–23 28340338 10.1016/j.cell.2017.02.007 PMC5432200

[15] Boija A 2018 Transcription factors activate genes through the phase-separation capacity of their activation domains Cell 175 1842–55 30449618 10.1016/j.cell.2018.10.042 PMC6295254

[16] Boeynaems S 2018 Protein phase separation: a new phase in cell biology Trends Cell Biol 28 420–35 29602697 10.1016/j.tcb.2018.02.004 PMC6034118

[17] Rhine K, Vidaurre V and Myong S 2020 RNA droplets Annu. Rev. Biophys 49 247–65 32040349 10.1146/annurev-biophys-052118-115508 PMC7695521

[18] Schuster BS 2020 Identifying sequence perturbations to an intrinsically disordered protein that determine its phase-separation behavior Proc. Natl Acad. Sci. USA 117 11421–31 32393642 10.1073/pnas.2000223117 PMC7261017

[19] Sawyer IA, Bartek J and Dundr M 2018 Phase separated microenvironments inside the cell nucleus are linked to disease and regulate epigenetic state, transcription and RNA processing Semin. Cell Dev. Biol 90 94–103 30017905 10.1016/j.semcdb.2018.07.001

[20] Uversky VN (2019). Intrinsically disordered proteins and their ‘mysterious’ (meta) physics. Front. Phys.

[21] Klosin A, Oltsch F, Harmon T, Honigmann A, Jülicher F, Hyman AA and Zechner C 2020 Phase separation provides a mechanism to reduce noise in cells Science 367 464–8 31974256 10.1126/science.aav6691

[22] Stroberg W and Schnell S 2018 Do cellular condensates accelerate biochemical reactions? Lessons from microdroplet chemistry Biophys. J 115 3–8 29972809 10.1016/j.bpj.2018.05.023 PMC6035290

[23] Wang Z, Potoyan DA and Wolynes PG 2018 Stochastic resonances in a distributed genetic broadcasting system: the NF κ B/I κ B paradigm J. R. Soc. Interface 15 20170809 29343631 10.1098/rsif.2017.0809 PMC5805984

[24] Peng A and Weber SC 2019 Evidence for and against liquid-liquid phase separation in the nucleus Non-coding RNA 5 50 10.3390/ncrna5040050 PMC6958436 31683819

[25] Wurtz JD and Lee CF 2018 Chemical-reaction-controlled phase separated drops: formation, size selection, and coarsening Phys. Rev. Lett 120 078102 29542937 10.1103/PhysRevLett.120.078102

[26] Pytowski L, Lee CF, Foley AC, Vaux DJ and Jean L 2020 Liquid-liquid phase separation of type II diabetes-associated IAPP initiates hydrogelation and aggregation Proc. Natl Acad. Sci. USA 117 12050–61 32414928 10.1073/pnas.1916716117 PMC7275713

[27] Lee CF, Brangwynne CP, Gharakhani J, Hyman AA and Jülicher F 2013 Spatial organization of the cell cytoplasm by position-dependent phase separation Phys. Rev. Lett 111 088101 24010479 10.1103/PhysRevLett.111.088101

[28] Zwicker D, Seyboldt R, Weber CA, Hyman AA and Jülicher F 2017 Growth and division of active droplets provides a model for protocells Nat. Phys 13 408–13

[29] Berry J, Weber SC, Vaidya N, Haataja M and Brangwynne CP 2015 Rna transcription modulates phase transition-driven nuclear body assembly Proc. Natl Acad. Sci. USA 112 E5237–45 26351690 10.1073/pnas.1509317112 PMC4586886

[30] Yamamoto T, Yamazaki T and Hirose T 2020 Phase separation driven by production of architectural RNA transcripts Soft Matter 16 4692–8 32396591 10.1039/c9sm02458a

[31] Gasior K, Zhao J, McLaughlin G, Forest MG, Gladfelter AS and Newby J 2019 Partial demixing of RNA–protein complexes leads to intradroplet patterning in phase-separated biological condensates Phys. Rev. E 99 012411 30780260 10.1103/PhysRevE.99.012411 PMC6739070

[32] Glotzer SC 1995 Computer simulations of spinodal decomposition in polymer blends Annu. Rev. Comput. Phys 2 1–46

[33] Tong C and Yang Y 2002 Phase-separation dynamics of a ternary mixture coupled with reversible chemical reaction J. Chem. Phys 116 1519–29

[34] Ilker E and Joanny J-F 2020 Phase separation and nucleation in mixtures of particles with different temperatures Phys. Rev. Res 2 023200

[35] Brangwynne CP, Tompa P and Pappu RV 2015 Polymer physics of intracellular phase transitions Nat. Phys 11 899–904

[36] Flory PJ 1942 Thermodynamics of high polymer solutions J. Chem. Phys 10 51–61

[37] Kepler TB and Elston TC 2001 Stochasticity in transcriptional regulation: origins, consequences, and mathematical representations Biophys. J 81 3116–36 11720979 10.1016/S0006-3495(01)75949-8 PMC1301773

[38] Alberts B (2018). Molecular Biology of the Cell.

[39] Caragine CM, Haley SC and Zidovska A 2019 Nucleolar dynamics and interactions with nucleoplasm in living cells Elife 8 e47533 31769409 10.7554/eLife.47533 PMC6879204

[40] Hohenberg PC and Halperin BI 1977 Theory of dynamic critical phenomena Rev. Mod. Phys 49 435

[41] Cahn JW and Hilliard JE 1958 Free energy of a nonuniform system. I. Interfacial free energy J. Chem. Phys 28 258–67

[42] Yang X, Zhao J, Wang Q and Shen J 2017 Numerical approximations for a three-component Cahn–Hilliard phase-field model based on the invariant energy quadratization method Math. Models Methods Appl. Sci 27 1993–2030

[43] Permann CJ (2020). MOOSE: enabling massively parallel multiphysics simulation. SoftwareX.

[44] Narita T, Yamamoto T, Hosoya E and Dobashi T 2003 Gibbs free energy expression for the system polystyrene in methylcyclohexane and its application to microencapsulation Langmuir 19 5240–5

[45] Yamamoto T, Narita T, Nobe M and Dobashi T 2004 Free energy of mixing for polymer solutions Macromolecules 37 3475–86

[46] Lin YT, Hufton PG, Lee EJ and Potoyan DA 2018 A stochastic and dynamical view of pluripotency in mouse embryonic stem cells PloS Comput. Biol 14 e1006000 29451874 10.1371/journal.pcbi.1006000 PMC5833290

[47] Hufton PG, Lin YT, Galla T and McKane AJ 2016 Intrinsic noise in systems with switching environments Phys. Rev. E 93 052119 27300842 10.1103/PhysRevE.93.052119

[48] Potoyan DA and Wolynes PG 2015 Dichotomous noise models of gene switches J. Chem. Phys 143 195101 26590554 10.1063/1.4935572 PMC4655464

[49] Kendon VM, Cates ME, Desplat J, Pagonabarraga I and Bladon P 2000 Inertial effects in three dimensional spinodal decomposition of a symmetric binary fluid mixture: a lattice Boltzmann study (arXiv:cond-mat/0006026)

[50] Zidovska A 2020 The self-stirred genome: large-scale chromatin dynamics, its biophysical origins and implications Curr. Opin. Genet. Dev 61 83–90 32497955 10.1016/j.gde.2020.03.008 PMC8164847

[51] Alshareedah I, Moosa MM, Raju M, Potoyan DA and Banerjee PR 2020 Phase transition of RNA−protein complexes into ordered hollow condensates Proc. Natl Acad. Sci. USA 117 15650–8 32571937 10.1073/pnas.1922365117 PMC7354941

[52] Kaur T, Raju M, Alshareedah I, Davis RB, Potoyan DA and Banerjee PR 2020 Sequence-encoded and composition-dependent protein−RNA interactions control multiphasic condensate topologies (10.1101/2020.08.30.273748) PMC7870978 33558506

[53] Lu T and Spruijt E 2020 Multiphase complex coacervate droplets J. Am. Chem. Soc 142 2905–14 31958956 10.1021/jacs.9b11468 PMC7020193

[54] Towns J 2014 Xsede: accelerating scientific discovery Comput. Sci. Eng 16 62–74

