# Supplementary material for: Liquid–liquid phase separation driven compartmentalization of reactive nucleoplasm
Source: Phys Biol. Author manuscript; Available in PMC 2021 Jun 14. (PMC8201646; doi:10.1088/1478-3975/abc5ad)
Supplement: Supplementary Information [file NIHMS1699554-supplement-Supplementary_Information.pdf]

Supporting Information for: "Liquid-liquid phase separation driven compartmentalization of reactive nucleoplasm."

Authors: Rabia Laghmach, Davit A. Potoyan

July 13, 2020

1 Supporting Information

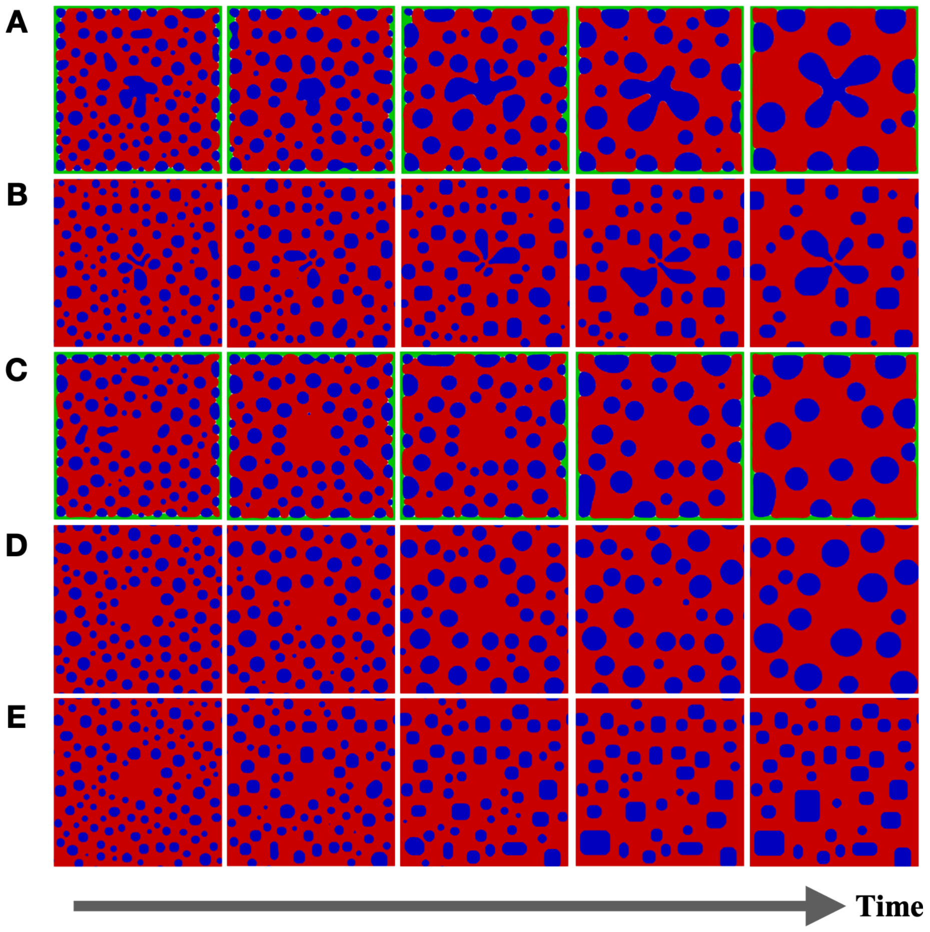

Figure 1: Evolution of phase-field variables φ_i for **a fast dynamical regime** $\tau_d = \tau$. From up to bottom: snapshots corresponding to simulation results with A) $\tau_R = \tau_P = \tau$; B) $\tau_P = 100\tau_R = 100\tau$; C) $\tau_R = 10\tau_P = 10\tau$; D) $\tau_R = \tau_P = 10\tau$; and E) $\tau_P = 10\tau_R = 100\tau$.

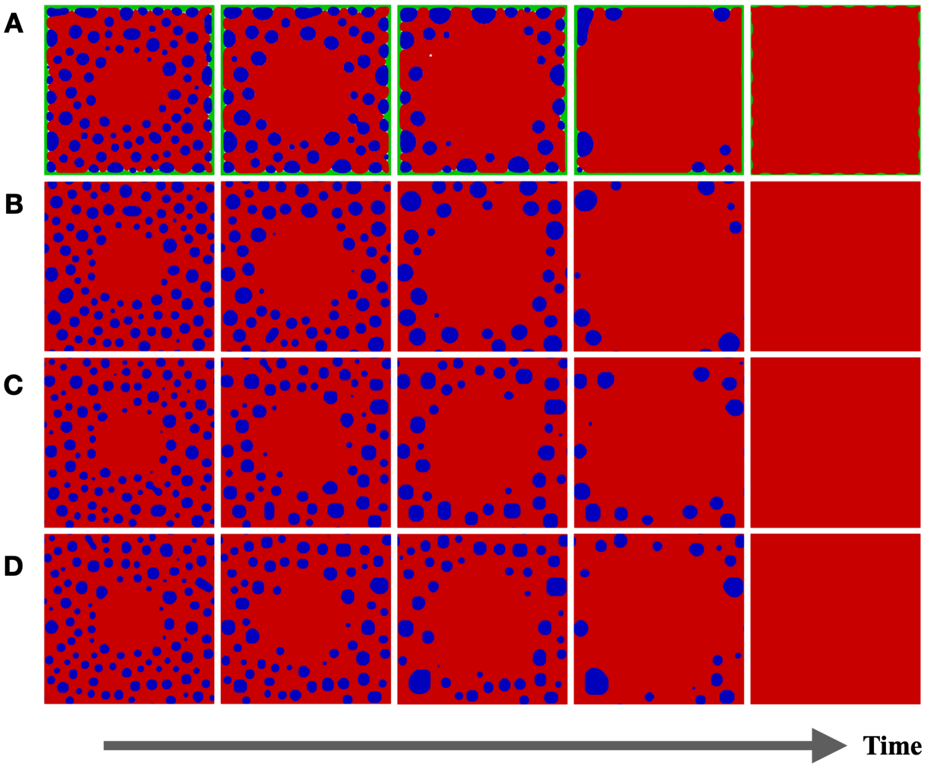

Figure 2: Evolution of phase-field variables φ_i for **a fast dynamical regime** $\tau_d = \tau$ **with** $\tau_R = 50\tau$. From up to bottom: snapshots corresponding to simulation results with A) $\tau_P = \tau$; B) $\tau_P = 10\tau$; C) $\tau_P = 50\tau$; and D) $\tau_P = 100\tau$.

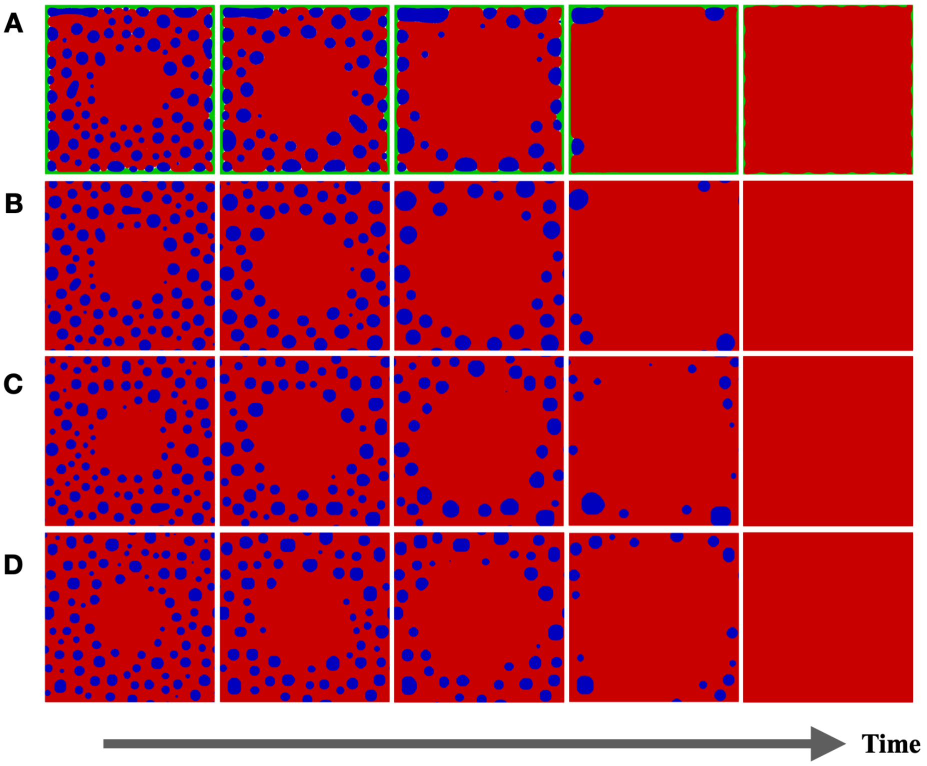

Figure 3: Evolution of phase-field variables φ_i for **a fast dynamical regime** $\tau_d = \tau$ **with** $\tau_R = 100\tau$. From up to bottom: snapshots corresponding to simulation results with A) $\tau_P = \tau$; B) $\tau_P = 10\tau$; C) $\tau_P = 50\tau$; and D) $\tau_P = 100\tau$.

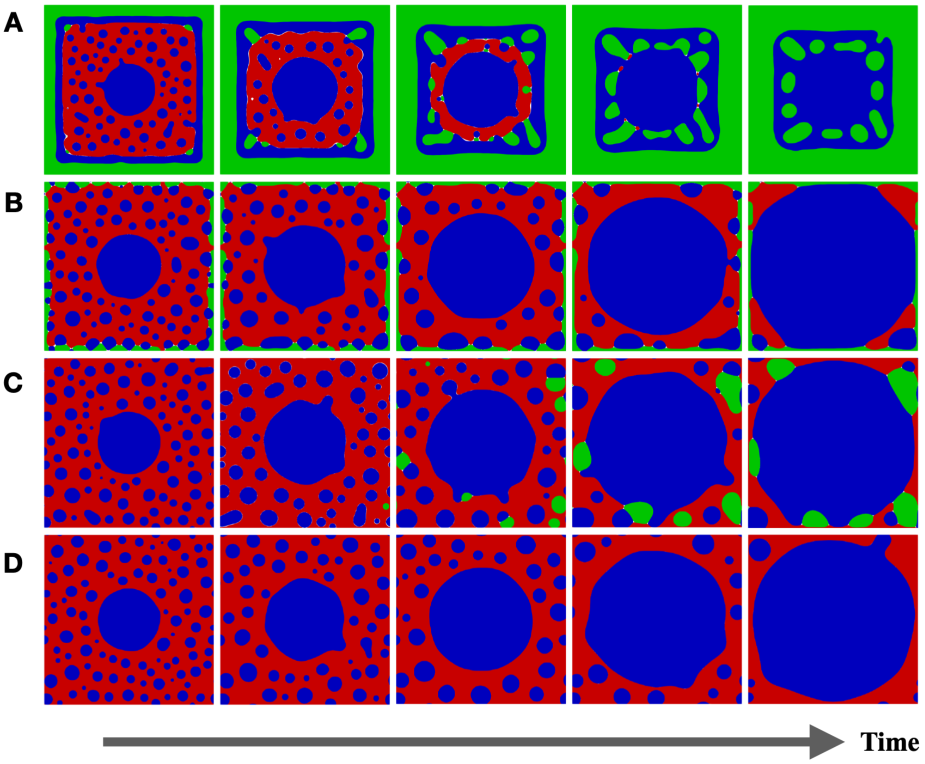

Figure 4: Evolution of phase-field variables φ_i for **an intermediate dynamical regime** $\tau_d = 10\tau$ **with** $\tau_R = \tau$. From up to bottom: snapshots corresponding to simulation results with A) $\tau_P = \tau$; B) $\tau_P = 10\tau$; C) $\tau_P = 50\tau$; and D) $\tau_P = 100\tau$.

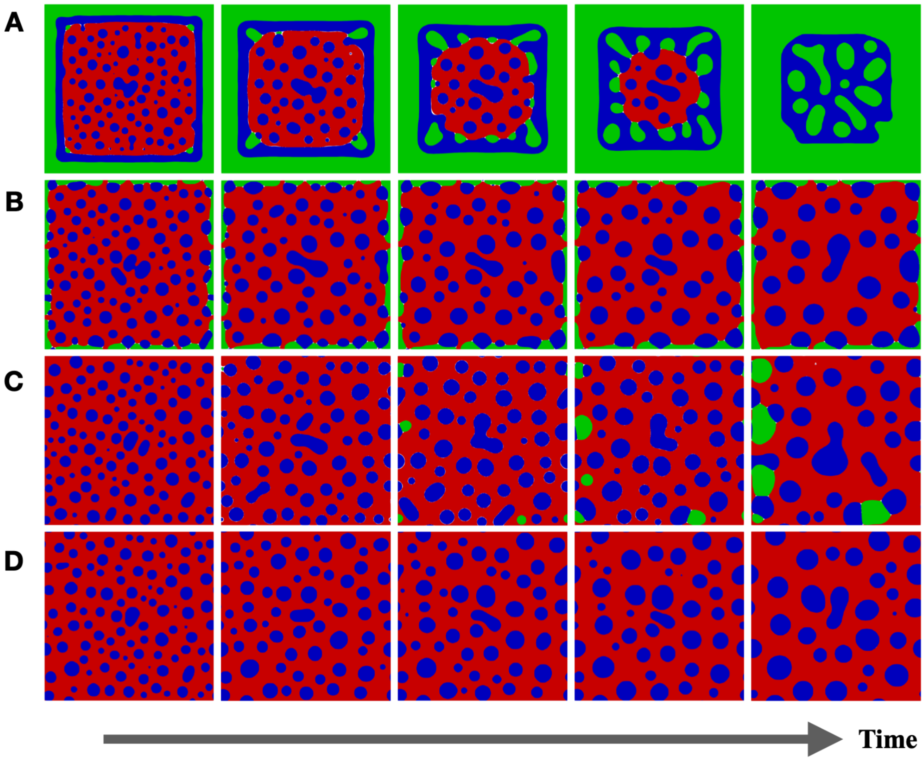

Figure 5: Evolution of phase-field variables φ_i for **an intermediate dynamical regime** $\tau_d = 10\tau$ **with** $\tau_R = 10\tau$. From up to bottom: snapshots corresponding to simulation results with A) $\tau_P = \tau$; B) $\tau_P = 10\tau$; C) $\tau_P = 50\tau$; and D) $\tau_P = 100\tau$.

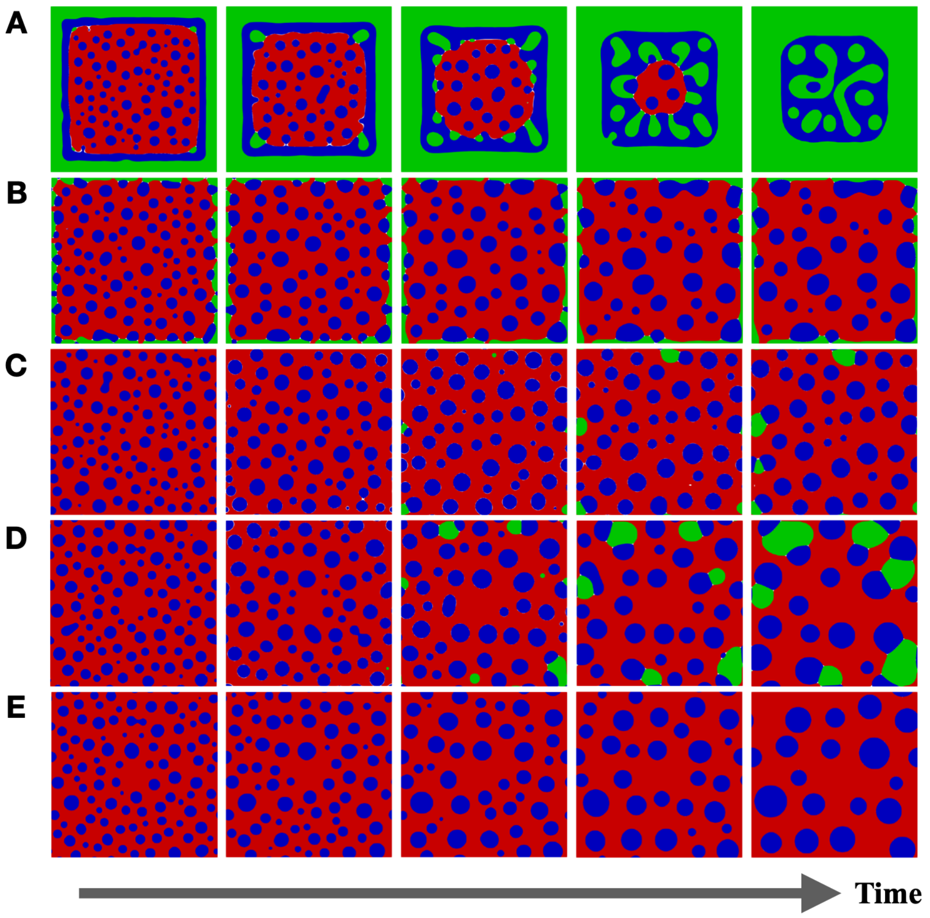

Figure 6: Evolution of phase-field variables φ_i for **an intermediate dynamical regime** $\tau_d = 10\tau$. From up to bottom: snapshots corresponding to simulation results with A) $\tau_R = 50\tau_P = 50\tau$; B) $\tau_R = 5\tau_P = 50\tau$; C) $\tau_R = \tau_P = 50\tau$; D) $\tau_R = 2\tau_P = 100\tau$; and E) $\tau_R = \tau_P = 100\tau$.

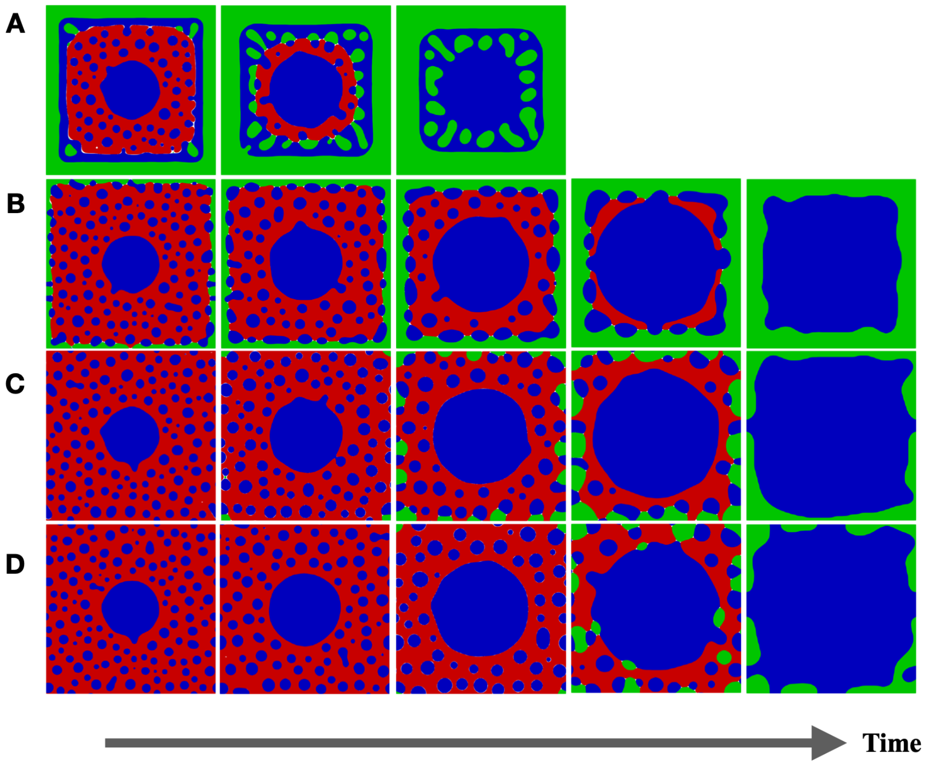

Figure 7: Evolution of phase-field variables φ_i for **a slow dynamical regime** $\tau_d = 100\tau$ **with** $\tau_R = \tau$. From up to bottom: snapshots corresponding to simulation results with A) $\tau_P = \tau$; B) $\tau_P = 10\tau$; C) $\tau_P = 50\tau$; and D) $\tau_P = 100\tau$.

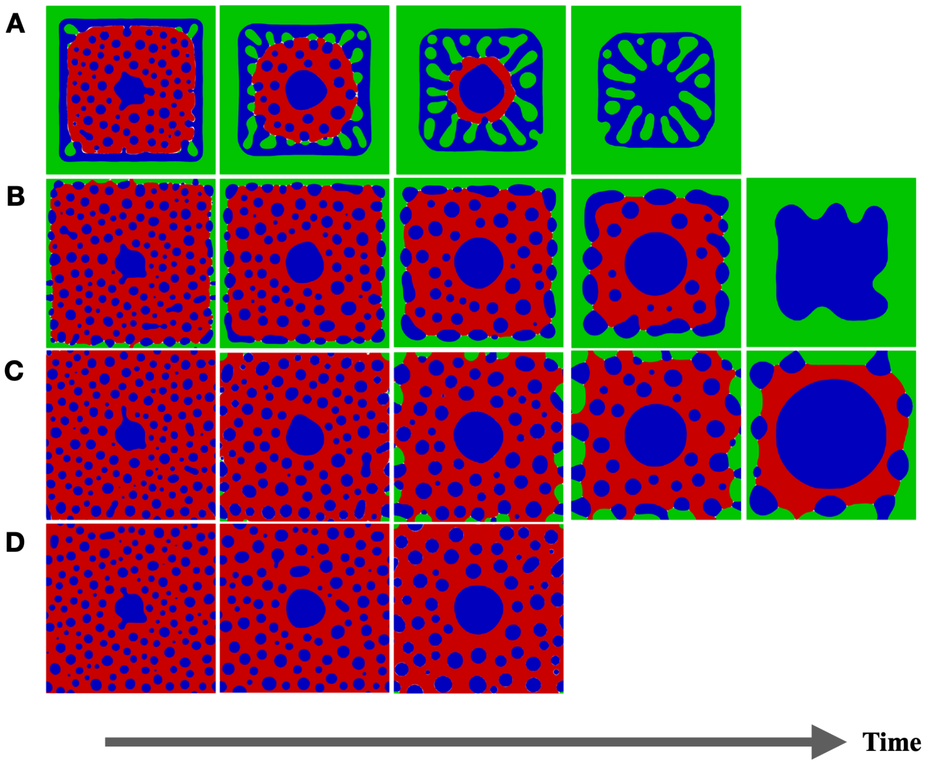

Figure 8: Evolution of phase-field variables φ_i for **a slow dynamical regime** $\tau_d = 100\tau$ **with** $\tau_R = 10\tau$. From up to bottom: snapshots corresponding to simulation results with A) $\tau_P = \tau$; B) $\tau_P = 10\tau$; C) $\tau_P = 50\tau$; and D) $\tau_P = 100\tau$.

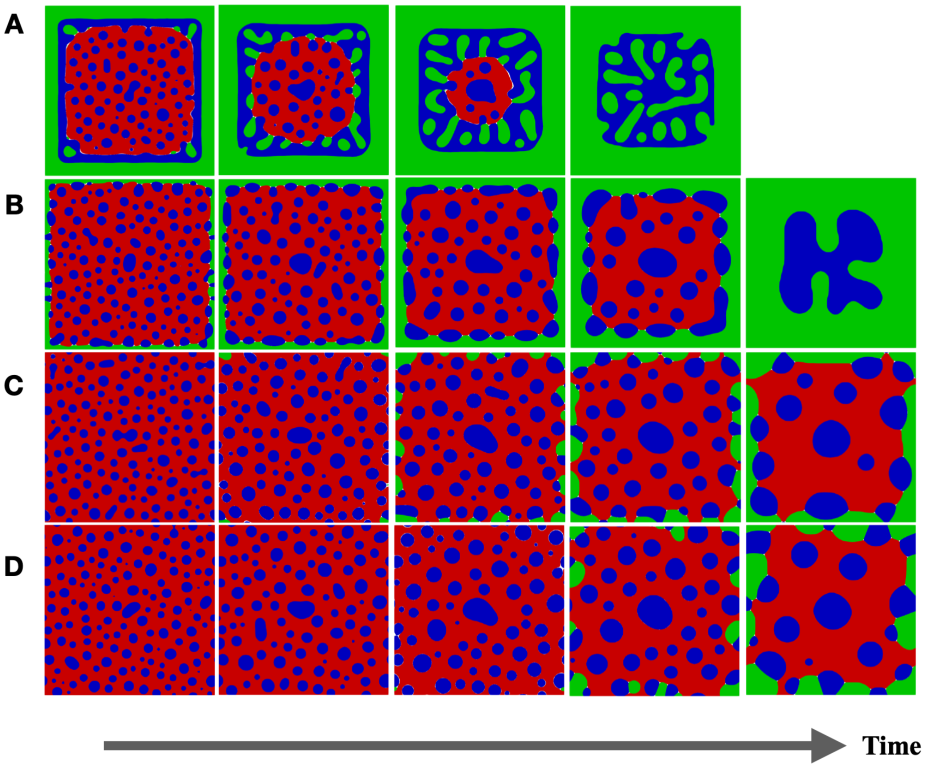

Figure 9: Evolution of phase-field variables φ_i for **a slow dynamical regime** $\tau_d = 100\tau$ **with** $\tau_R = 50\tau$. From up to bottom: snapshots corresponding to simulation results with A) $\tau_P = \tau$; B) $\tau_P = 10\tau$; C) $\tau_P = 50\tau$; and D) $\tau_P = 100\tau$.

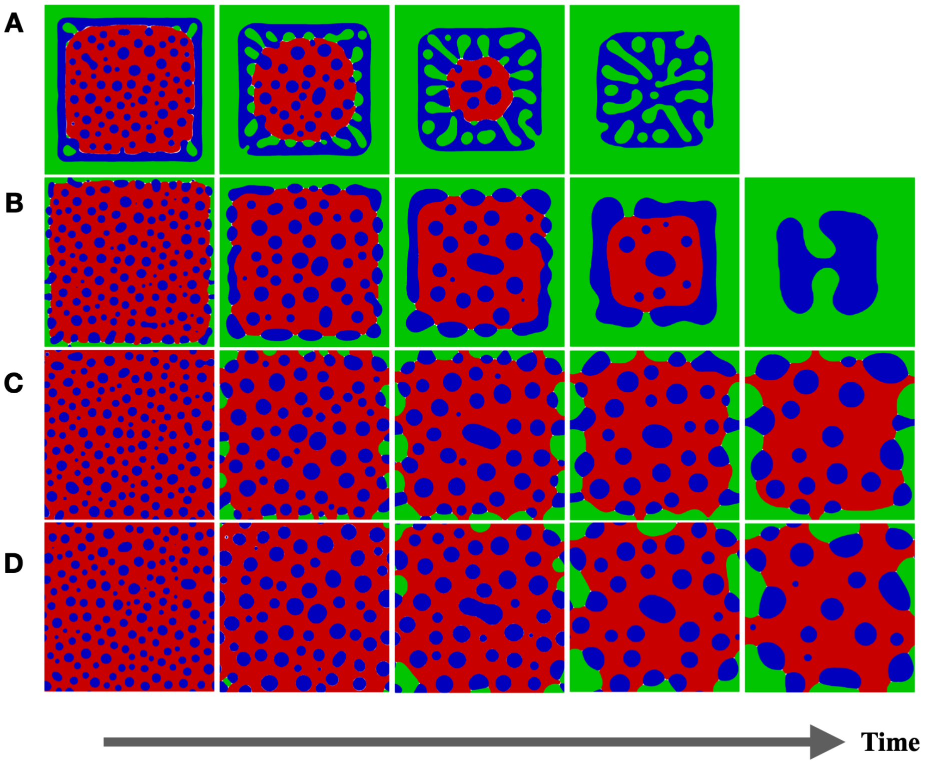

Figure 10: Evolution of phase-field variables φ_i for **a slow dynamical regime** $\tau_d = 100\tau$ **with** $\tau_R = 100\tau$. From up to bottom: snapshots corresponding to simulation results with A) $\tau_P = \tau$; B) $\tau_P = 10\tau$; C) $\tau_P = 50\tau$; and D) $\tau_P = 100\tau$.

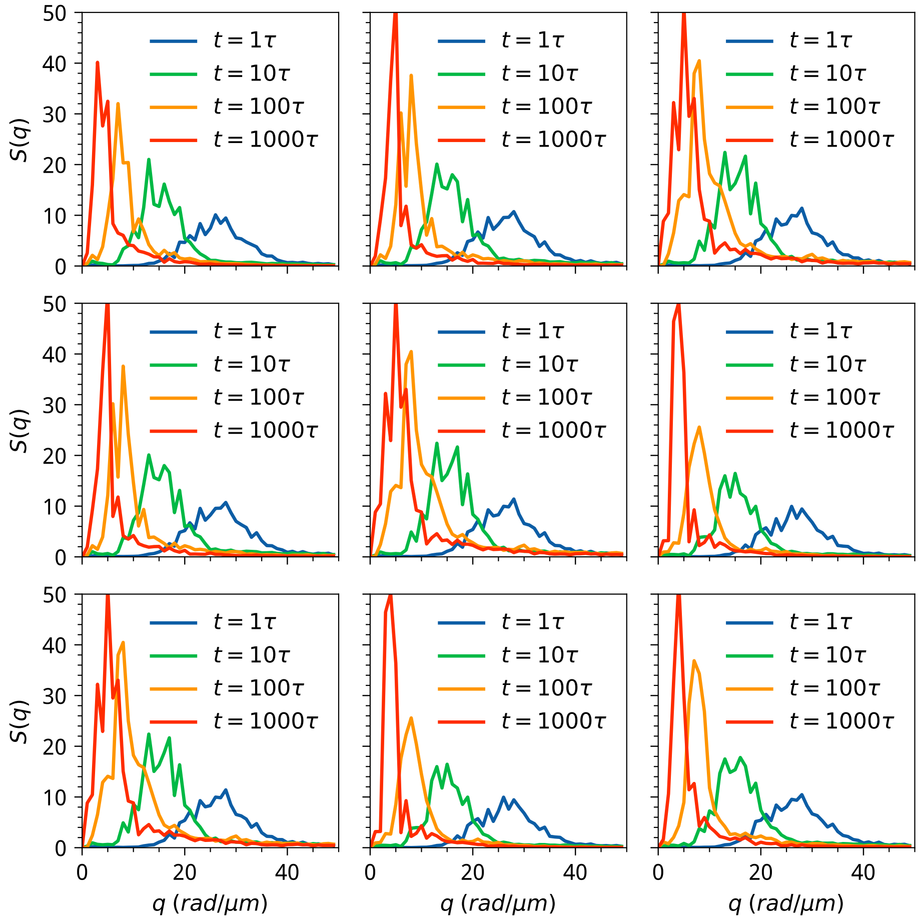

Figure 11: Structure factors for a fast dynamical regime $\tau_d = \tau$. Three panels corresponds to (A) Fixed transcription rate $\tau_R = 1$, varying translation from left to right: $\tau_P = \{1, 10, 100\}\ \tau$ (B) Fixed transcription rate $\tau_R = 10\tau$, varying translation from left to right: $\tau_P = \{1, 10, 100\}\ \tau$ (C) Fixed transcription rate $\tau_R = 100\tau$, varying translation from left to right: $\tau_P = \{1, 10, 100\}\ \tau$.

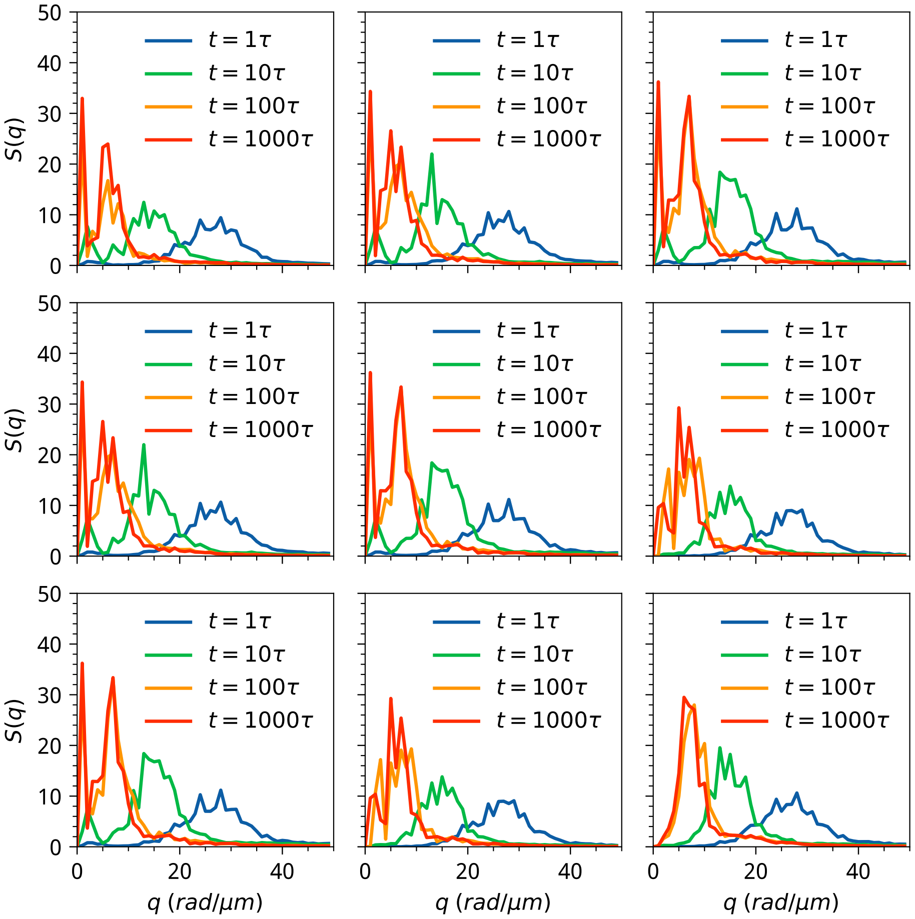

Figure 12: Structure factors for an intermediate dynamical regime $\tau_d = 10\tau$. Three panels corresponds to (A) Fixed transcription rate $\tau_R = \tau$, varying translation from left to right: $\tau_P = \{1, 10, 100\}\,\tau$ (B) Fixed transcription rate $\tau_R = 10\tau$, varying translation from left to right: $\tau_P = \{1, 10, 100\}\,\tau$ (C) Fixed transcription rate $\tau_R = 100\tau$, varying translation from left to right: $\tau_P = \{1, 10, 100\}\,\tau$.

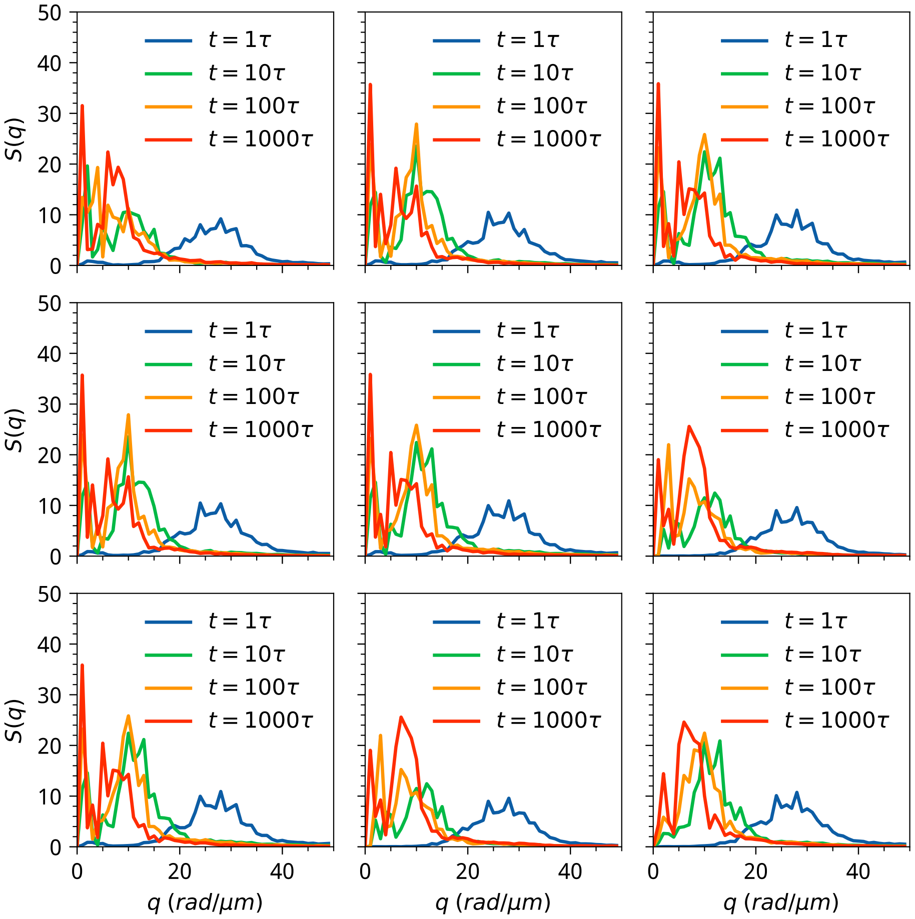

Figure 13: Structure factors for a slow dynamical regime $\tau_d = 100\tau$. Three panels corresponds to (A) Fixed transcription rate $\tau_R = \tau$, varying translation from left to right: $\tau_P = \{1, 10, 100\}\,\tau$ (B) Fixed transcription rate $\tau_R = 10\tau$, varying translation from left to right: $\tau_P = \{1, 10, 100\}\,\tau$ (C) Fixed transcription rate $\tau_R = 100\tau$, varying translation from left to right: $\tau_P = \{1, 10, 100\}\,\tau$.

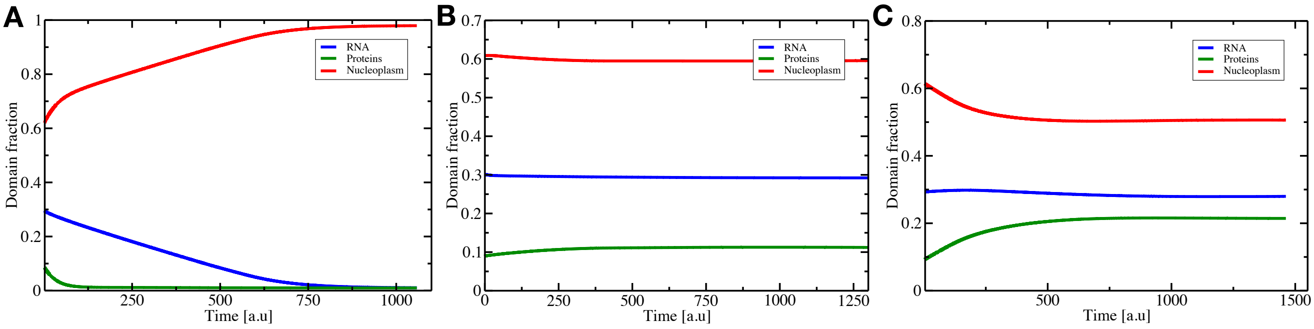

Figure 14: Temporal evolution of the average concentration of the three species $< \varphi_i >$ at different kinetic regimes with $\tau_R = \tau_P = 100\tau$: A) fast dynamical regime $\tau_d = \tau$; B) an intermediate dynamical regime $\tau_d = 10\tau$; C) slow dynamical regime $\tau_d = 100\tau$.
